# Unusual Manifestation of Extrapulmonary Tuberculosis: A Soft Tissue Mass in an Immunocompetent Patient

**DOI:** 10.7759/cureus.91060

**Published:** 2025-08-26

**Authors:** Myint B Thu, Ni Ni Lwin, Wut Y Hlaing, Norah H Aung, Tomoko Ikuine

**Affiliations:** 1 Internal Medicine, NYC Health + Hospitals/Harlem, New York, USA; 2 Internal Medicine, University of Medicine (1) Yangon, Yangon, MMR; 3 Internal Medicine, Harlem Hospital Center, Macomb, USA; 4 Public Health, Western Illinois University, Macomb, USA; 5 Sleep Medicine, Hennepin Healthcare, Minneapolis, USA

**Keywords:** disseminated tuberculosis, extrapulmonary tuberculosis (eptb), mycobacterium tuberculosis infection, tb, tuberculoma

## Abstract

We report a case of disseminated tuberculosis (TB) manifesting as a soft tissue mass of the back associated with pain and difficulty walking. Its clinical presentation poses diagnostic challenges and can sometimes mimic neoplasms. The first imaging study with computed tomography was suggestive of metastatic bone disease or locally advanced malignancy. Advanced imaging studies, such as magnetic resonance imaging (MRI) or computed tomography (CT), are required to evaluate the extent of organ involvement. A definitive diagnosis necessitates histopathological analysis and microbiological confirmation through acid-fast Bacilli staining and polymerase chain reaction (PCR) testing for *Mycobacterium tuberculosis *(MTB). Disseminated TB is rare in immunocompetent patients, which accounts for <2% of all TB cases. It usually occurs from progressive primary infection or the reactivation of latent infection focus through lymphohematogenous spread. The risk of communicable diseases has increased due to recent migration, so it is important to investigate MTB in patients with unusual presentations, especially for people from endemic areas.

## Introduction

According to the WHO, the estimated incidence of *Mycobacterium tuberculosis* (MTB) globally in 2022 was 133 cases per 100,000 population. In the U.S., Alaska ranks first and New York ranks fourth in the incidence of TB. Tuberculosis is mainly transmitted by aerosol droplets. Disseminated TB is defined as two or more non-contiguous sites of TB infection resulting from lymphohematogenous spread [1].

TB is primarily considered a pulmonary infection, but it can be spread to other organs via hematogenous or lymphogenous routes. Most TB-infected patients contain the tuberculous form of TB and are presented as a latent granuloma form by the adaptive immune response. The latter is activated to resurface as a primary active infection at one site or spread to distant organs after years of contact with TB [2].

Even with the available treatment, the diagnosis of TB still poses a challenge. As the study performed in India, 243 (5.8%) of 4219 post-neonatal autopsied studies over 29 years were found to have disseminated TB [3]. Also, a systematic review and meta-analysis by Gupta found that 87.9% of patients who had a co-infection of HIV were noted to have disseminated TB even though the patients were not diagnosed with tuberculosis [4].

Disseminated TB can present with a variety of symptoms, making a prompt diagnosis challenging. Thus, we should have a high degree of suspicion in both high TB incidence and low TB incidence areas, where it is expected to be deceptive in getting the definitive diagnosis. This case highlights the challenges in diagnosing disseminated TB.

## Case presentation

**Figure 1:**
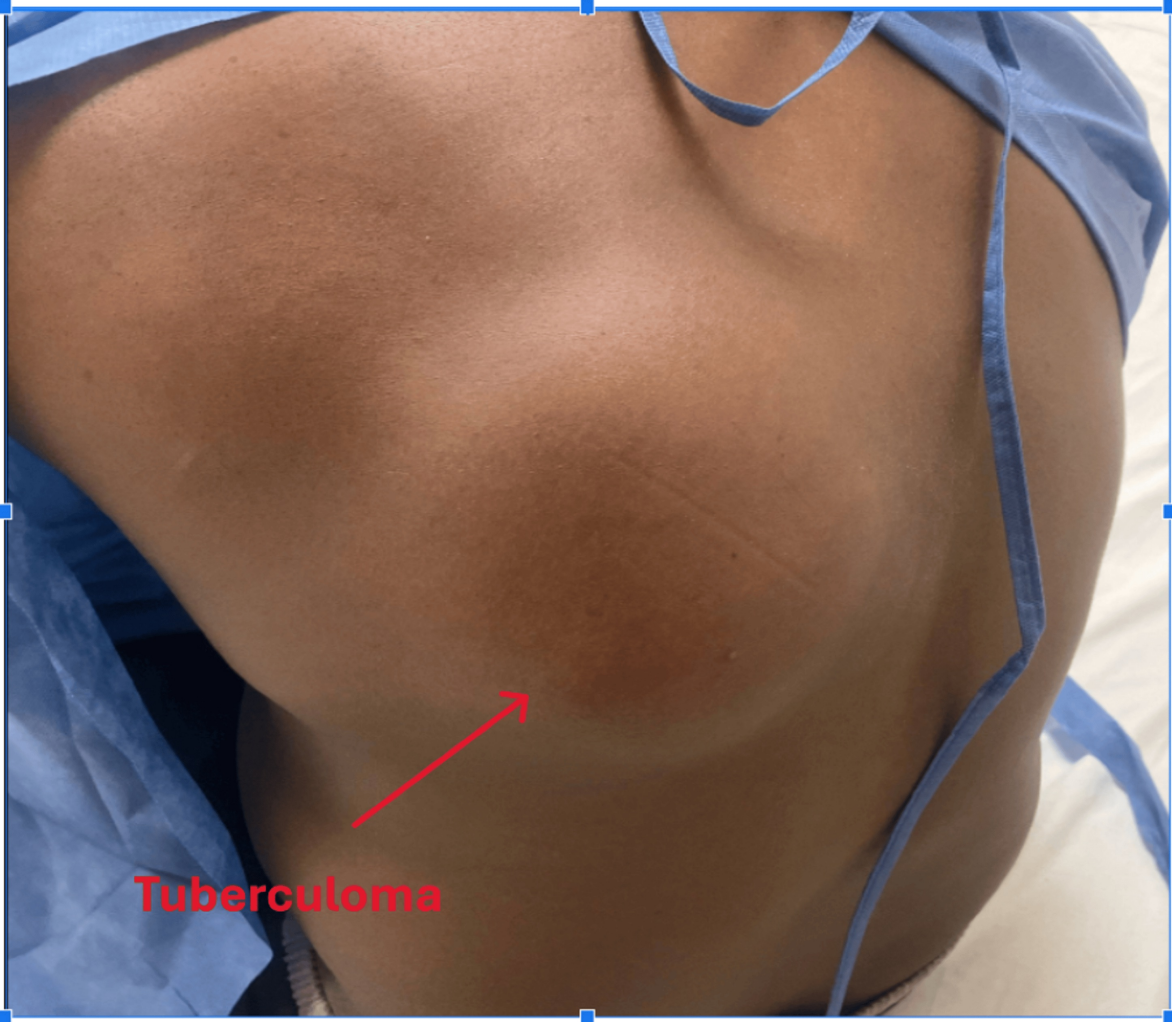
Tuberculoma of the right upper back

A 42-year-old man from Guinea presented to the emergency department with a complaint of difficulty walking for two weeks. This symptom was preceded by progressive worsening of upper back pain that had been ongoing for six months and a mass on his upper back noted for the last two months. The patient described his back pain as sharp and intermittent, radiating to the neck and lower back along the vertebral line. It was accentuated by touch or pressure and accompanied by numbness in the legs, with an unstable gait. He also reported experiencing subjective fever and chills. He had a past medical history of persistent hypokalemia and hypertension. He was taking methocarbamol 500 mg nightly and acetaminophen 500 mg 6 hourly for the pain. He denied any smoking, drinking alcohol, or using drugs. He migrated from Guinea two years ago with an unknown history of TB contact. The initial blood pressure was 169/116 mmHg, the heart rate was 112 bpm, the temperature was 98.8 ℉, and the oxygen saturation was 98%. On physical examination, a 10 x 7 cm soft and tender mass was noted on the right upper back without any sign of inflammation (Figure 1).

**Figure 2:**
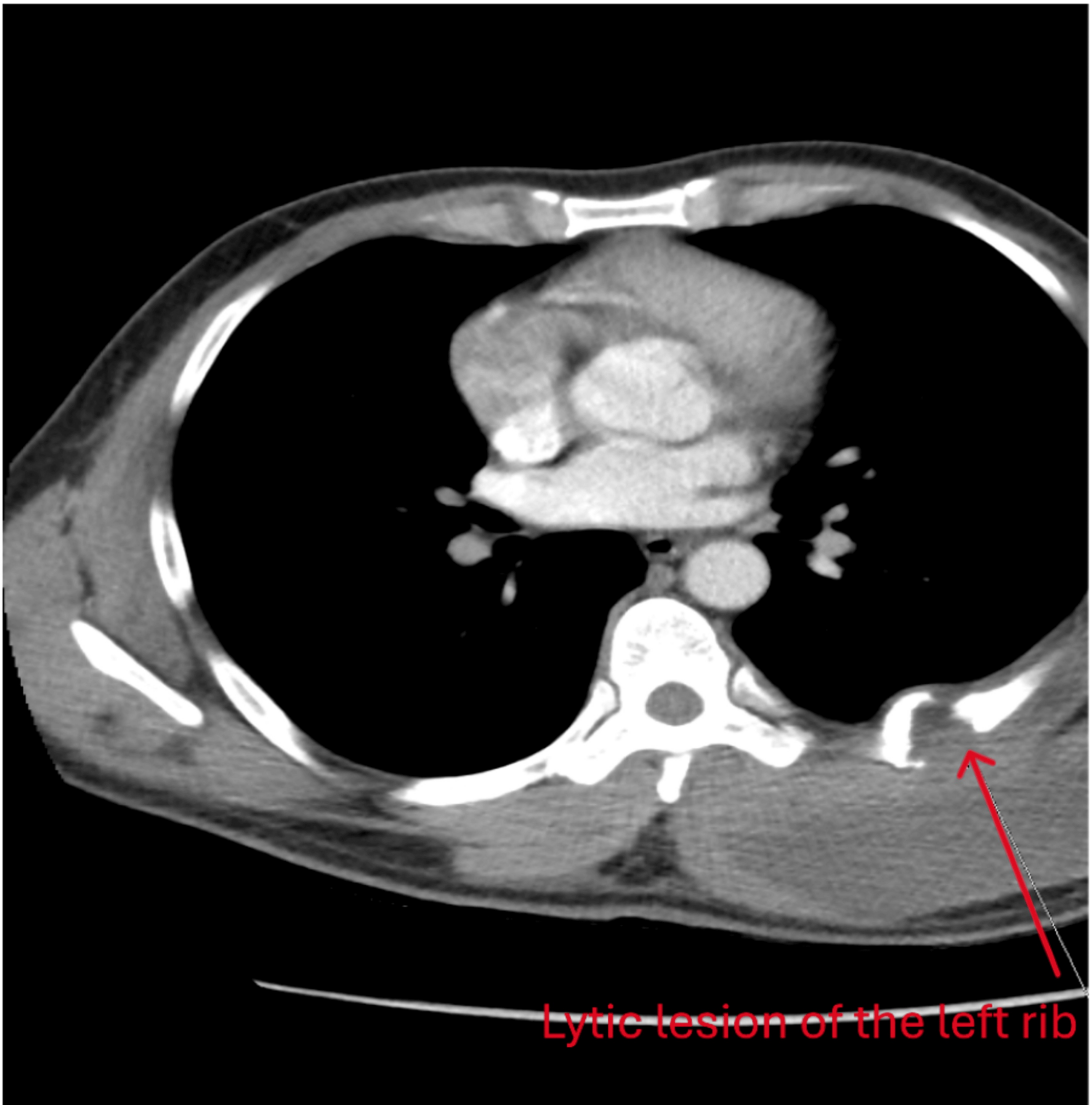
CT of the chest showing a lytic lesion of the rib due to TB

The neurological evaluation revealed a motor strength of 4/5 in the lower extremities and 5/5 in the upper extremities. The lower part of the body below the T4 dermatome level was numb, and gait instability was noted. The rest of the physical findings were insignificant. Initial laboratory studies were not significant, except for hypokalemia. The hypokalemia is refractory, despite daily potassium replacement. Further workup was undertaken for hyperadrenalism and renal tubular acidosis or tubular damage. Findings of interest were metabolic alkalosis with alkaline urine pH 7.5, microscopic hematuria, urine anion gap 54, and aldosterone-renin ratio of 8.5 ng/dL. We commenced losartan and later added spironolactone for hypertension. The CT scan of the thoracic spine and chest showed an intramuscular mass of 12x5.4x11.2 cm (Figure 2) in the lateral latissimus dorsi and erector spinae with multiple lytic lesions in the ribs and spines, iliac bones, and another fluid-filled lesion on the lateral right first rib.

**Figure 3:**
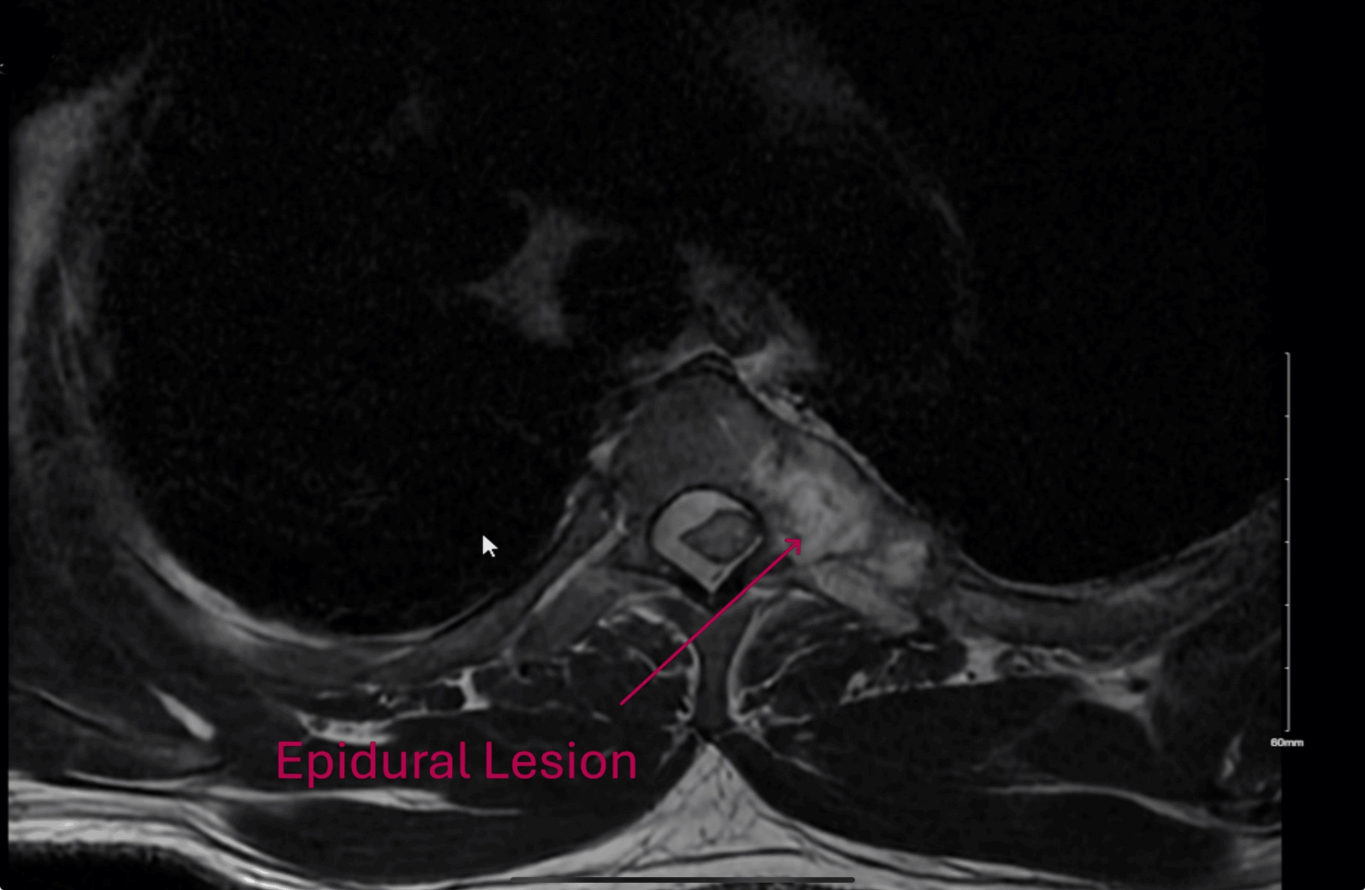
MRI of the thoracic spine showing the epidural lesion

MRI of the whole spine showed hypointense epidural lesions extending from the C2-C3 through the T4 level, which caused severe narrowing of the spinal canal and indentation of the cord. At the T3 level, the lesion encased the epidural spaces (Figure 3).

Additional labs revealed C-reactive protein (CRP) 43.0 mg/dl, erythrocyte sedimentation rate (ESR) 82 mm/hr, and no monoclonal bands in immunofixation (Table 1). The free light chain Kappa and lambda ratio was 9.10 with no monoclonal bands, the angiotensin-converting enzyme (ACE) level was normal, and the Quantiferon TB gold test was positive. The HIV screening test was negative. The soft tissue lesion was aspirated, which was about 115 ml, with acute and chronic inflammation cells, including macrophages, without any malignant cells. Bone marrow biopsy revealed normal marrow, flow cytometry was negative, and MTB PCR from the abscess drainage fluid was positive. The mycobacterium was not resistant to antibiotics, including rifampin, isoniazid, pyrazinamide, and ethambutol (RIPE therapy) was initiated. The neurosurgeon carried out a T2-4 laminectomy that involved the removal of the epidural lesion. A biopsy of the epidural lesion revealed a reactive lymph node with large noncaseating and caseating granuloma without any indication of malignancy. The patient's sensory symptoms improved within a few days, but the motor symptoms persisted during the hospital stay. The patient had a prolonged hospital stay due to his social issue, but no postoperative complications occurred. A repeat spine MRI after one and a half months showed interval development of an abscess in the T3 inferior endplate to the T6 spinous process level. We performed needle aspiration at the bedside but did not send for additional microbiological testing. The patient was subsequently discharged back to the community with follow-up with primary care and infectious disease.

**Table 1:** Laboratory values

Lab	Results	Normal value
Renin activity	0.905 ng/ml/hr	0.167-5.380 ng/ml/hr
Aldostreone	7.5 ng/dl	<=23.2 ng/dl
Aldosterone-Renin Ratio	8.5	-
C-Reactive Protein	43.0 mg/dl	0.0-5.0 mg/L
Sedimentation Rate	82 mm/hr	0-15 mm/hr
Kappa	243.81 mg/L	<=8.99 mg/L
Lambda	26.79 mg/L	<=6.99
Kappa: Lambda Ratio	9.10	0.70-6.02
Angiotensin-Converting Enzyme Level	43 U/L	14-82 U/L
Immunoglobulin G	1939 mg/dl	610-1660 mg/dl
Immunoglobulin M	83 mg/dl	35-242 mg/dl
Immunoglobulin A	278 mg/dl	84-499 mg/dl
Immunofixation Urine	No monoclonal band	-
Protein Electrophoresis	Normal electrophoresis pattern	-

## Discussion

TB is a bacterial infection caused by *Mycobacterium tuberculosis* that can affect every organ, but mainly involves the pulmonary system. In our case, the patient presented with a painful soft tissue mass of the upper back associated with a weakness of the lower extremities, emphasizing the uncommon occurrence of extrapulmonary TB manifestations.

Soft tissue tuberculosis, caused by *Mycobacterium tuberculosis*, is a rare extrapulmonary infection that mostly affects young and middle-aged people and is somewhat more common in men [5]. As our patient did not have signs of pulmonary infection, cryptic tuberculosis was difficult to diagnose. It could be due to either activation of a latent infection, healing of the primary focus, or a missed pulmonary TB diagnosis in the past [6]. A delay in diagnosing soft tissue tuberculosis may exacerbate the disease, increase TB transmission, and hasten the evolution of antibiotic resistance. 

Extrapulmonary tuberculosis is more common in elderly people and those with immunocompromised conditions such as diabetes, HIV/AIDS, chronic renal disease, malignancies, or those on immunosuppressive therapy [7]. However, our patient did not have any comorbidities, except that he was a high-risk patient from the endemic area. In addition, in this patient, despite receiving appropriate treatment, new tuberculous abscesses appeared after one month, and the existing ones increased in size. This paradoxical reaction, in which the appearance of a new tuberculous abscess after starting treatment has been reported, can also occur in immunocompetent patients [8-10].

The unexplained recurrent mild hypokalemia in our patients despite replacement was investigated for endocrine causes and tubular defects. There were case reports of refractory hypokalemia associated with disseminated TB or multidrug-resistant TB [11]. These may be due to the disseminated TB causing tubular defects [12]. After treatment with losartan and spironolactone, the electrolyte imbalance was resolved.

There is no definitive guideline for extrapulmonary TB. In general, the drug-susceptible disseminated TB management is the same as for pulmonary TB, which is RIPE for six months. However, individualization of the treatment may be warranted [13]. In our case, the repeated MRI after one and a half months of RIPE showed new abscesses, so we planned to continue the RIPE regimen for one year. 

## Conclusions

Extrapulmonary tuberculosis can be difficult to diagnose due to its varied and often subtle presentations, especially in immunocompetent individuals. It is important to rule out tuberculosis in subtle clinical presentations of patients from endemic regions, even in the absence of pulmonary symptoms. To receive timely treatment and intervention, early identification, appropriate imaging, and histological diagnosis are crucial. The complexity of disseminated TB is further demonstrated by the paradoxical progression following treatment and refractory hypokalemia brought on by possible tubular dysfunction. Tailored management of prolonged RIPE therapy may be needed in severe or uncommon instances.
